# Single-Cell Imaging of Mitochondrial Metabolism and Remodeling in C2C12 Murine Skeletal Muscle Cells upon Differentiation

**DOI:** 10.3390/ijms27114689

**Published:** 2026-05-22

**Authors:** Rozhin Penjweini, Alessandra Pasut, Branden Roarke, Katie A. Link, Dan L. Sackett, Jay R. Knutson

**Affiliations:** 1 Laboratory for Advanced Microscopy and Biophotonics, National Heart, Lung, and Blood Institute (NHLBI), National Institutes of Health (NIH), Building 10, Room 5D14, Bethesda, MD 20892-1412, USA; rozhin.penjweini@nih.gov (R.P.); katie.link@nih.gov (K.A.L.); 2 Laboratory of Angiogenesis and Vascular Metabolism, KU Leuven, Herestraat 49, 3000 Leuven, Belgium; 3 Cytoskeletal Dynamics Group, Division of Basic and Translational Biophysics, *Eunice Kennedy Shriver* National Institute of Child Health and Human Development (NICHD), National Institutes of Health (NIH), Building 29B, Room 1E10, Bethesda, MD 20892-0924, USA; sackettd@mail.nih.gov

**Keywords:** mitochondrial metabolism, mitochondrial morphology and dynamics, single-cell imaging

## Abstract

As primary sites for oxygen consumption and energy production via oxidative phosphorylation (OXPHOS), mitochondria play a central role in the regulation of bioenergetics and generation of key metabolic intermediates for myogenic cell growth. Common methods to study mitochondria and their metabolism typically rely on population-level analyses, which can mask potential differences in individual cells. In this study, we used various imaging approaches to investigate the interplay between intracellular oxygenation, mitochondrial metabolism and dynamics in a model of myogenic differentiation. Fluorescence imaging of intracellular oxygen revealed that myogenic differentiation is accompanied by progressive shifts in intracellular oxygenation that depend upon and reflect changes in mitochondrial metabolism (i.e., higher oxygen consumption and adenosine triphosphate (ATP) production). By measuring intracellular oxygenation, we showed that mitochondrial metabolism reduces oxygen availability in the cytosol and the nucleus. Real-time redox imaging at the single-cell level further highlighted substantial metabolic heterogeneity and a shift toward OXPHOS as differentiation progressed. Morphological analyses revealed that during myogenic differentiation, mitochondria increase in size while becoming less mobile and overlapping less with microtubules. Overall, this study illustrates the value of combining complementary imaging approaches to provide a comprehensive single-cell perspective on mitochondrial metabolism, remodeling and spatial organization during myogenesis.

## 1. Introduction

Myogenesis is characterized by extensive metabolic changes that support the energetic needs and requirements for biomass production as muscle cells proliferate and differentiate [1]. Mitochondria are the major organelles involved in energy production via cellular respiration [2,3]. Beyond adenosine triphosphate (ATP) synthesis, mitochondria also generate key metabolic intermediates and biosynthetic precursors for myogenic cell growth. Changes in morphology and interactions with other organelles allow mitochondria to adjust and respond to different stressors and metabolic needs, to effectively support myogenesis [3,4,5]. 

The C2C12 murine skeletal muscle cell line is an immortalized mouse myoblast cell line capable of differentiating in culture, upon appropriate treatment. This process involves the fusion of replicating myoblast cells into a post-mitotic multinucleated syncytium, structurally reorganizing into a myotube. Due to high energetic demands, the need for increased mitochondrial activity in myogenic differentiation has been well documented [6]. It is known that C2C12 cells shift from a highly glycolytic state in proliferative conditions to relying predominantly on oxidative phosphorylation (OXPHOS) upon differentiation—and this phenomenon requires dramatic remodeling of the mitochondrial network [6,7,8]. Current approaches to investigate cell and mitochondrial metabolism typically rely on bulk population measurements that mask differences at the individual cell level [9,10]. Therefore, how mitochondria support the metabolic needs of myogenic cells in different myogenic cell states remains poorly understood [3,8].

Recent advances in biophysical technologies and metabolic imaging approaches, including via genetically encoded fluorescent biosensors, have enabled the study of metabolism at the single and subcellular level [10,11,12]. As several studies have shown, cell heterogeneity, including at the metabolic level, becomes clear when examined at sufficiently high resolution [1,10].

In this study, we used multiple single-cell-based imaging techniques to investigate intracellular oxygenation, as well as mitochondrial metabolism and dynamics (morphology and motion) in C2C12 muscle cells, before and after differentiation. These time points were chosen as they represent clearly distinct cellular states with well-defined changes in cell metabolism (at least at the population level). These conditions enabled us to test our imaging techniques in a more controlled environment, which also allowed us to obtain more robust data and avoid potential confounding factors. In addition, mitochondria in proliferative myoblasts were challenged with OXPHOS inhibitor or uncoupler. High-glucose (HG) vs. glucose-starved (GS) media were used to investigate the effects of mitochondrial O_2_ consumption on cell differentiation, intracellular (cytosolic and nuclear) pO_2_ level as well as metabolic redox ratio. In situ intracellular oxygen partial pressure (pO_2_) was assessed via fluorescence lifetime imaging microscopy (FLIM) of a Förster resonance energy transfer (FRET)-based oxygen sensor [11,13]. We also exploit the autofluorescence lifetime imaging of the metabolic co-factors nicotinamide adenine dinucleotide (NAD(P)H) and flavin adenine dinucleotide (FAD^+^) to reveal changes in cellular redox ratio. The remodeling of mitochondrial morphology upon differentiation was revealed using Mitochondrial Network Analysis (MiNA) and the measurement of mitochondrial shape. Changes in mitochondrial association with microtubules upon differentiation were also investigated. Mitochondrial directed vs. random motions were investigated with temporal and spatiotemporal image correlation spectroscopy [14]. We couple these single-cell measurements with complementary mitochondria targeted bulk population measurements, including membrane potential and reactive oxygen species (ROS, superoxide) generation. Additionally, we look at absolute ATP production. Collectively, this combination of single-cell and multi-cell measurements was analyzed together to build a better understanding of how mitochondria support metabolic needs of myogenic cells in different states.

With single-cell based imaging techniques, we show that: (i) In contrast to proliferative C2C12 cells that rely primarily on glycolysis for their metabolic needs, differentiated C2C12 cells are highly metabolically active and rely heavily on mitochondrial O_2_ consumption for OXPHOS. (ii) Further, we show changes in mitochondrial O_2_ consumption due to exogenous mitochondrial uncoupler and inhibitor, glucose starvation and different media-imposed O_2_. These changes influence nuclear and cytosolic pO_2_ level, as well as mitochondrial ATP and ROS production. (iii) Combining measurements of pO_2_ with measurements of metabolic co-factors, NAD(P)H and FAD^+^, links mitochondrial O_2_ consumption to functional metabolic changes. (iv) As differentiation progresses, mitochondrial size increases, they become more round and mitochondrial connectivity and motions decrease. (v) Finally, our single-cell analyses show substantial cell-to-cell variability among proliferative cells, which reduces during transit from single to multinucleated states.

## 2. Results and Discussion

### 2.1. Single-Cell-Based Imaging of Intracellular Oxygenation and Characterization of Mitochondrial Metabolism During Myogenic Differentiation

**Figure A1:**
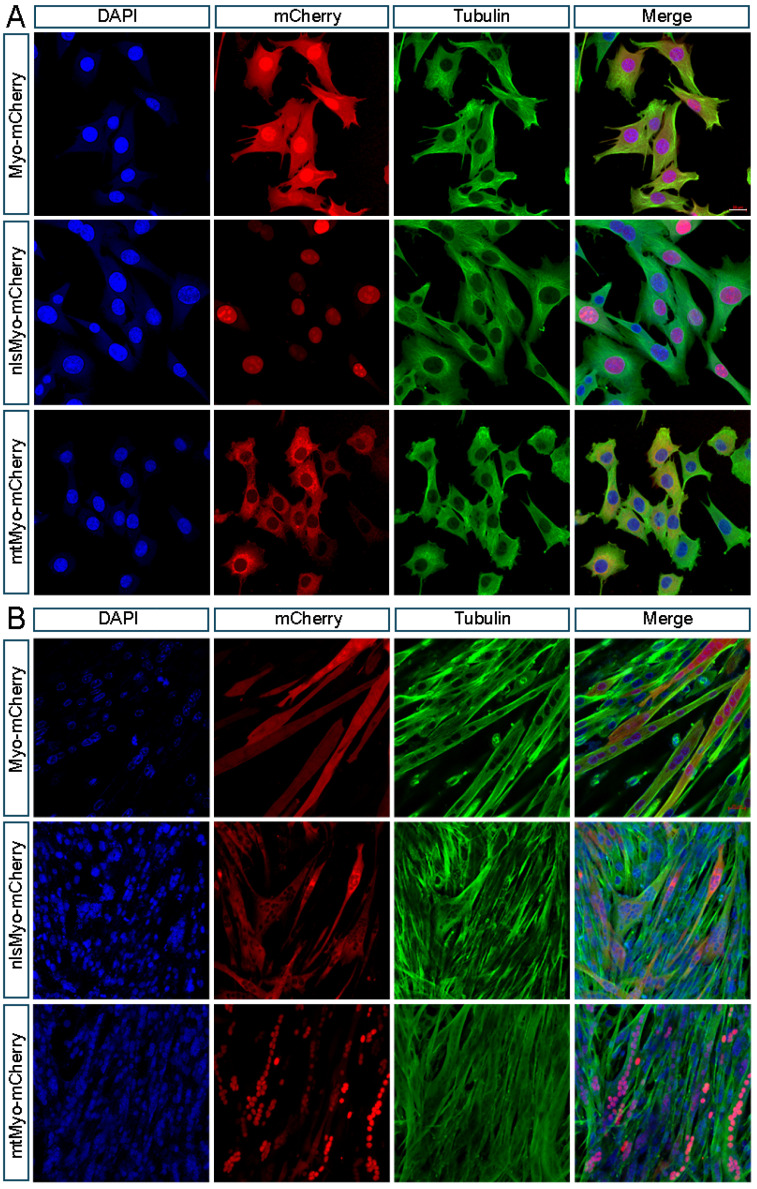
(**A**), representative immunofluorescence images of C2C12 cells transfected with Myo-mCherry targeted to the cytosol (Myo-mCherry), nuclei (nlsMyo-mCherry) and mitochondria (mt Myo-mCherry), cultured in standard/growth medium. (**B**), representative immunofluorescence images of C2C12 cells cultured in differentiation medium. mCherry signal is shown in red, tubulin in green. Nuclei were counterstained with DAPI (blue). Scale bar is 20 μm.

**Figure A2:**
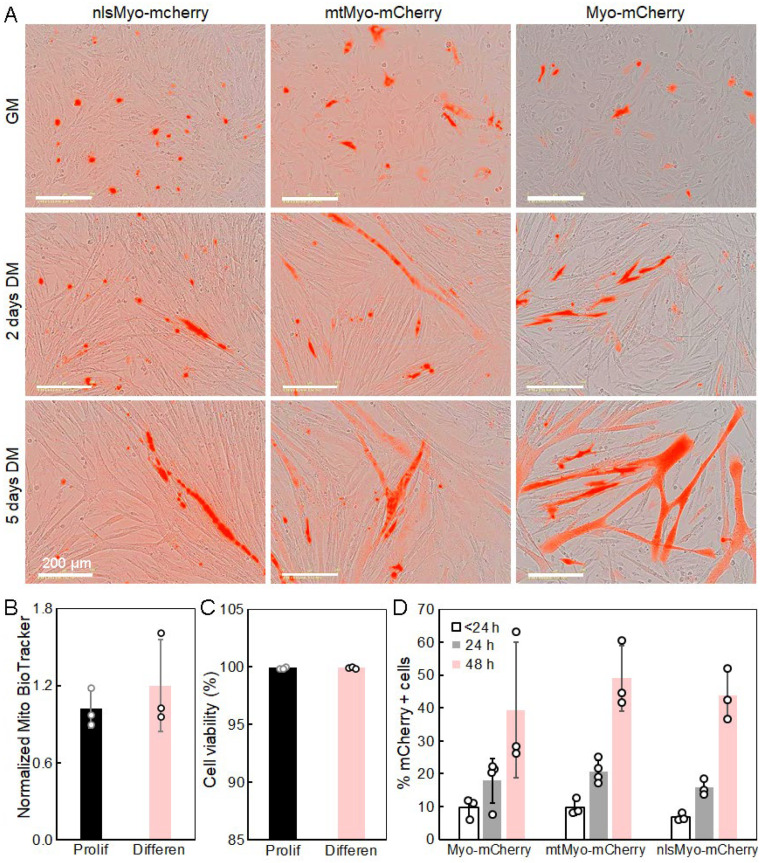
Cytotoxicity and transfection studies of Myo-mCherry. (**A**), images of proliferative (Prolif) and differentiated (Differen) C2C12 cells cultured in growth media (GM) or differentiation media (DM) and transfected with Myo-mCherry targeted to the nuclei (nlsMyo-mCherry), mitochondria (mtMyo-mCherry) or cytosol (Myo-mCherry). Cytotoxicity studies using (**B**), the BioTracker 488 Green Mitochondria Dye and (**C**), the Cell Viability Imaging Kit. (**D**), percentage of mCherry + C2C12 cells over total DAPI+ cells, calculated at 3 different time points post-transfection, before 24 h (<24 h), at 24 h and 48 h. Data represents mean value  ±  standard deviation from 3–4 independent transfection experiments.

Measurements of O_2_ consumption using isolated mitochondria and bulk analyses of proliferative cells show that C2C12 cells mainly rely on glycolysis for ATP production. However, during differentiation myogenic cells switch to OXPHOS to meet the cells’ increased energy demand [6,15]. Ultimately, the differentiation of skeletal muscle cells is highly dependent on mitochondrial energy supply and accelerated mitochondrial function during differentiation [16,17,18]. Cell-to-cell heterogeneity is of interest, both for correlation with other phenomena and to avoid common experimental and analytical pitfalls. Here, we use fluorescence lifetime imaging of our previously reported O_2_ sensor, Myo-mCherry, to evaluate pO_2_ changes in the intramitochondrial and intracellular (nuclear and cytosolic) environment in C2C12 at the single-cell level [11,13]. Briefly, Myo-mCherry is a Förster resonance energy transfer (FRET)-probe composed of the nonfluorescent acceptor, myoglobin, which can accept energy from the fluorescent donor, mCherry. Oxygenated and deoxygenated myoglobin have different absorption profiles and therefore different FRET efficiencies. To increase the spatial resolution of the probe, C2C12 cells were transfected with three different versions of the Myo-mCherry sensor, targeting the mitochondria, nucleus and cytosol (whole cell). The correct localization of the three probes was confirmed via immunofluorescence of the mCherry signal (Figure A1 in Appendix A.1). Cell viability studies using the MitoBioTracker and Cell Viability Assay showed no cytotoxicity of the oxygen probe (Figure A2 in the Appendix A.2). The role of mitochondrial O_2_ consumption in the regulation of intracellular pO_2_ and transition to differentiation was also investigated by challenging cells with glucose starvation (GS), OXPHOS inhibitor (rotenone) and uncoupler (DNP).

When switched to differentiation media, proliferative C2C12 cells fused to multinucleated cells within 48–72 h unless exposed to DNP, rotenone or GS, in agreement with previous studies [15,16,17]. This shows that mitochondrial activity, O_2_ consumption and active complex I are required for proper differentiation.

**Figure 1:**
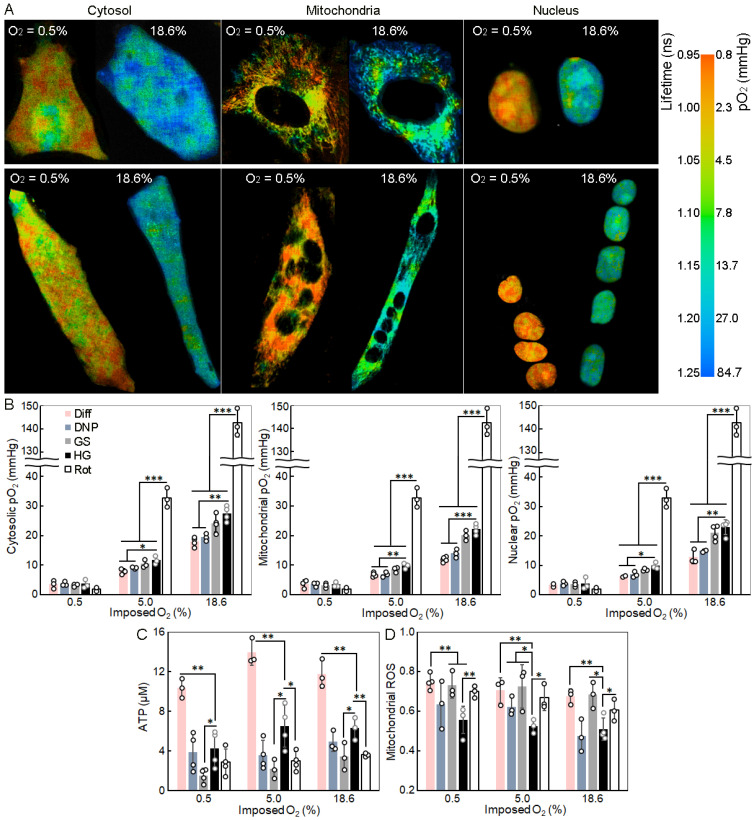
Imaging of intracellular oxygenation in C2C12 cells. (**A**), pseudo color mapping of oxygen partial pressure (pO_2_) in the cytosol, mitochondria and nuclei of proliferative and differentiated C2C12 in response to imposed O_2_% of 0.5% (left side) and 18.6% (right side). Red indicates lower pO_2_ values, whereas blue indicates higher pO_2_ values. (**B**), quantification of cytosolic, mitochondrial and nuclear pO_2_ in C2C12 cells in different conditions. (**C**), ATP production and (**D**), mitochondrial ROS at different imposed O_2_ (%) in C2C12 cells. All samples were labeled with Hoechst to normalize both ATP and ROS measurements based on the amount of DNA present in each well. Error bars indicate standard deviations. For each two independent groups, statistical significance was defined using *t*-test *p* < 0.05 (95% confidence level). Statistical significance was shown with stars on each plot: three stars (***) for *p*-value < 0.001, two stars (**) for *p*-value < 0.01 and one star (*) for *p*-value < 0.05. Each data point (empty circles) represents an independent experiment.

Figure 1A shows pseudo color mapping of cytosolic, intramitochondrial and nuclear Myo-mCherry lifetime and their corresponding pO_2_ distribution in proliferative (top row) and differentiated (bottom row) C2C12 cells at 0.5% (left side) and 18.6% (right side) imposed O_2_, with a shift toward red indicating lower lifetime and pO_2_ values, and blue indicating higher values. As expected, the intracellular pO_2_ measured with Myo-mCherry in both proliferative and differentiated cells directly correlated to the imposed O_2_%.

As quantified in Figure 1B, the transition from proliferative (HG, black bars) to differentiated (Diff, pink bars) C2C12 cells was accompanied by a decrease in cytosolic, mitochondrial and nuclear pO_2_ levels, at constant imposed pO_2_, indicating an increase in respiration rate and OXPHOS occurring during differentiation across all compartments. Our data also show lower pO_2_ in the mitochondria as compared to the cytoplasm, providing evidence that mitochondria are the primary sites for O_2_ consumption in muscle cells. We observed that pO_2_ was low in the nucleus, likely due to the perinuclear localization of the mitochondria affecting nuclear pO_2_ (Figure 1B). Uncoupling of mitochondrial respiratory activity (DNP, blue bars) in proliferative cells resulted in a lower level of compartmental pO_2_ as compared to cells kept in a regular media (HG, black bars), consistent with the expected uncoupler-induced increase in O_2_ consumption. In contrast, the inhibition of mitochondrial complex I by rotenone in proliferative cells (Rot, white bars) increased subcellular pO_2_. The decrease in cytosolic and nuclear pO_2_ levels in DNP-treated proliferative cells and the increase in pO_2_ levels in rotenone-treated proliferative cells demonstrates the dependence of these compartmental pO_2_ levels on mitochondrial O_2_ consumption.

**Figure A3:**
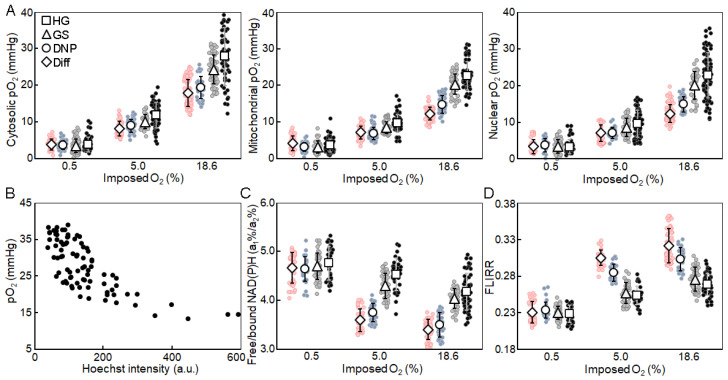
Heterogeneity in cellular metabolism. (**A**), scatter plots of cytosolic, mitochondrial and nuclear oxygen partial pressure (pO_2_) in individual proliferative cells treated with high-glucose (HG), glucose-starved (GS), uncoupler DNP and upon differentiation (Diff). (**B**), scatter plot of cytosolic pO_2_ versus Hoechst intensity in HG cells at O_2_ = 18.6%, showing the heterogeneity due to the cell cycle. Scatter plots of (**C**), free/bound NAD(P)H and (**D**), fluorescence lifetime-based redox ratio (FLIRR) in individual cells. Error bars represent standard deviation.

Scatter plots of pO_2_ in individual cells (see Figure A3A in the Appendix A.3) showed a significant cell-to-cell variability. This heterogeneity was more pronounced among proliferative cells kept in high-glucose (standard) media compared to all other conditions. Single-cell correlation of cytosolic pO_2_ versus Hoechst intensity (see Figure A3B in the Appendix A.3) showed that the heterogeneity in pO_2_ is due to contributions from cells in various stages of the cell cycle. In agreement with a previous study [19], while O_2_ consumption and OXPHOS (causing lower cytosolic pO_2_) were most prominent during G2/M phase, proliferative cells in HG media rely more on glycolysis for their energy needs in G1 phase. Differentiated cells enter and remain non-dividing and in G1 phase of the cell cycle and glucose-starved (GS) cells are all in G1 phase. Therefore differentiated and GS cells show lower heterogeneity in intracellular pO_2_ than proliferative cells, which contain all phases of the cell cycle. 

As shown in Figure 1C,D, in addition to changes in the intracellular pO_2_ level, rotenone treatment in proliferative cells led to decreased overall ATP and to increased mitochondrial ROS (superoxide) at all imposed O_2_%. Conversely, treatment of proliferative cells with DNP did not significantly change overall the ATP or mitochondrial ROS at an imposed O_2_ of 18.6%. However, in an environment with a limited O_2_ supply (5% and 0.5%), treatment of proliferative cells with DNP caused substantial disruption in ATP production, as shown by low overall ATP and high levels of mitochondrial ROS (superoxide). Glucose starvation (GS) in proliferative cells slightly but not significantly decreased intracellular pO_2_ level and resulted in a decrease in overall ATP detected and an increase in mitochondrial ROS (superoxide) at all imposed O_2_% compared to cells in high-glucose media (Figure 1C,D). These results indicate that at least in our tested conditions, in glucose-starved C2C12 cells, fatty acid oxidation (FAO) is either not engaged and/or only partially engaged. In agreement, a previous study also shows that oxidative metabolism is not increased in C2C12 cultured under low or no glucose (Figure 1) [15]. In addition, glucose starvation (5.6 mM) was found to stimulate the expression of glucose transporter protein type 1 (GLUT1), hexokinase 1 (HK1), glycolytic enzymes and autophagy. Overall, this suggests that FAO may not be the preferred metabolic adaptation in glucose-starved C2C12 cells [20].

As expected, differentiated C2C12 cells showed increased overall ATP and mitochondrial ROS when compared to proliferative cells in standard media (Figure 1C,D). All samples were additionally labeled with Hoechst to normalize the ATP and ROS measurements based on the amount of DNA present in each well.

We performed whole-cell proteomics to identify protein expression changes associated with differentiation, shown in Table A1 (Appendix A.4). Our data show that *α*- and *β*- subunits of ATP synthase are both increased in differentiated cells, as is citrate synthase. Since citrate synthase is often used as a marker of mitochondrial mass [21], these results suggest mitochondrial content is increased upon differentiation. Our proteomics analyses also showed that during C2C12 differentiation, desmin (a type III intermediate filament protein specific to differentiated muscle cells) progressively replaced vimentin (type III intermediate filament proteins), more commonly expressed in proliferative cells [22,23].

### 2.2. Single-Cell Imaging of Redox Metabolism in Myogenic Cells

While we showed that intracellular pO_2_ level can be used to infer the metabolic state of the cells, lifetime imaging of the metabolic co-factors NAD(P)H and FAD^+^ (as a proxy to NAD^+^) was performed to independently monitor the activity of OXPHOS vs. glycolytic metabolism in proliferative and differentiated cells.

NAD(P)H and FAD^+^ are endogenously fluorescent metabolic co-enzymes that exist in a free and in a protein-bound state, depending on the metabolic state of the cells. The relative amounts of the free/bound NAD(P)H can be measured via FLIM, and the ratio of the populations of free/bound NAD(P)H and FAD^+^ reflect changes in the metabolic state of the cells [24].

Here, we used the NAD(P)H free/bound fluorescence as an independent imaging approach to investigate redox metabolism in myogenic cells. The measured NAD(P)H signal reflects the balance between free/enzyme bound NAD(P)H across cytosolic and mitochondrial metabolic pathways. While several metabolic pathways can influence the NAD(P)H free/bound fluorescence signal, it is generally accepted that this reflects glycolysis vs OXPHOS. Generally, in cells with functioning mitochondria and in well-defined states as in the model described here (C2C12 before and after differentiation), a higher fraction of free NAD(P)H and lower contribution of bound NAD(P)H is a characteristic of glycolytic cells and vice versa.

**Figure 2:**
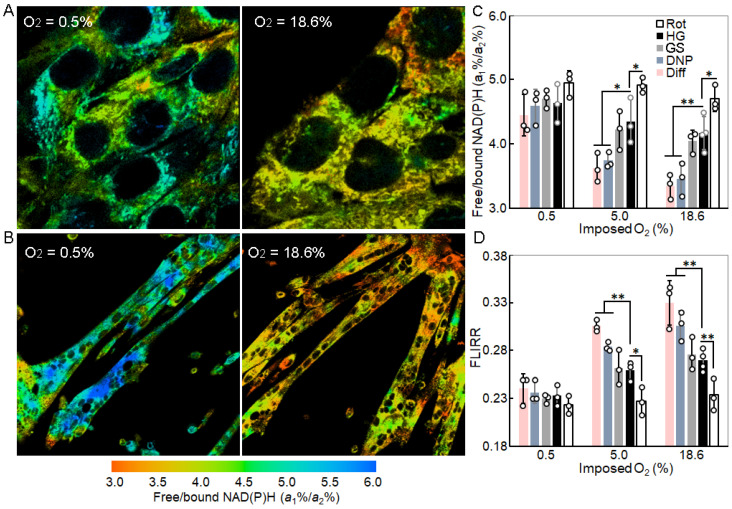
Metabolic redox imaging in C2C12 cells. Pseudo color mapping of free/bound NAD(P)H in the intracellular environment of (**A**), proliferative and (**B**), differentiating C2C12 cells in response to imposed O_2_ of 18.6% (left) and 0.5% (right); red indicates low free/bound NAD(P)H values, whereas blue indicates high free/bound NAD(P)H values. (**C**,**D**), quantification of free/bound NAD(P)H and fluorescence lifetime redox ratio (FLIRR) in C2C12 cultured under different conditions. Error bars indicate standard deviations. For each two independent groups, statistical significance was defined using *t*-test *p* < 0.05 (95% confidence level). Statistical significance was shown with stars on each plot: two stars (**) for *p*-value < 0.01 and one star (*) for *p*-value < 0.05. Each data point (empty circles) represents an independent experiment on a minimum of 20–30 cells.

Figure 2A,B show pseudo color mapping of free/bound NAD(P)H ratios in proliferative and differentiated C2C12 cells in response to imposed O_2_% of 0.5% (left side) and 18.6% (right side), with a shift toward red indicating lower NAD(P)H ratios and blue indicating higher NAD(P)H ratios. The effect of mitochondrial O_2_ consumption on free/bound NAD(P)H ratio was further studied by enhancing or inhibiting O_2_ consumption, modulating glucose and external O_2_ availability. As shown in Figure 2C, free/bound NAD(P)H ratios varied significantly in response to these tested conditions. A lower ratio of free/bound NAD(P)H was observed in proliferative cells treated with the mitochondrial uncoupler DNP, reflecting the anticipated increase in O_2_ consumption. Inhibition of mitochondrial electron transport chain activity using rotenone decreased mitochondrial conversion of NAD(P)H to NAD^+^ and increased free/bound NAD(P)H (Figure 2C). Glucose starvation (GS, gray bars) in proliferative cells did not have a significant impact on the ratio of free/bound NAD(P)H as compared to cells kept in a regular media (HG, black bars) (Figure 2C). Finally, the ratio of free/bound NAD(P)H was lower in metabolically active differentiated cells, probably because OXPHOS accounted for significant energy and ATP production (Figure 2C). These results indicate that differentiated muscle cells have lower glycolysis and more active mitochondria capable of higher O_2_ consumption compared to their proliferative counterpart.

As an additional measure of the cell metabolic state, we also assessed the fluorescence lifetime redox ratio or FLIRR (*a*_2_,_NAD(P)H %_/*a*_1_,_FAD+ %_). FLIRR was introduced by A. Periasamy [12] as a more accurate measure of the metabolic redox ratio compared to optical redox ratio (ORR; NAD(P)H/FAD^+^ ratio), with the latter dependent on laser power, optics and many other parameters that can cause miscalculation of the redox ratio [25].

As quantified in Figure 2D, at biologically relevant (5%) and atmospheric (18.6%) imposed O_2_, differentiated cells show higher FLIRR than proliferative cells, indicating an increase in OXPHOS upon differentiation. FLIRR also increased when proliferative cells were treated with DNP, but in this case, the increase in FLIRR no longer reflects a shift toward OXPHOS and rather the uncoupling effect of DNP. As expected, the highest anaerobic glycolysis was accompanied by the lowest FLIRR in the cells treated with rotenone. Glucose starvation in proliferative cells did not have a significant impact on FLIRR.

Overall, these results are consistent with the findings obtained with the myoglobin-mCherry sensor and support the conclusion that differentiated muscle cells have more active mitochondria, capable of higher O_2_ consumption, compared to their proliferative counterparts. FLIM analysis at single-cell resolution also reveals heterogeneity in the free/bound NAD(P)H ratio and FLIRR (or metabolic signature). This heterogeneity was more pronounced among the proliferative cells kept in high-glucose standard media (see scatter plots in Figure A3C,D in the Appendix A.3).

### 2.3. Changes of Mitochondrial Membrane Potential, Dynamics and Association with Microtubules During C2C12 Myogenesis

Mitochondrial dynamics play a crucial role in virtually all developmental processes [26]. We showed that differentiation of C2C12 cells is associated with an increase in mitochondrial O_2_ consumption and OXPHOS metabolism. As mitochondrial dynamics are specifically connected to developmental functions and differentiation [8,26], we investigated whether the remodeling of mitochondrial morphology and motion occurs in response to changes in cellular metabolism, especially the regulation of glycolysis–oxidative respiration shifts in our C2C12 model.

**Figure 3:**
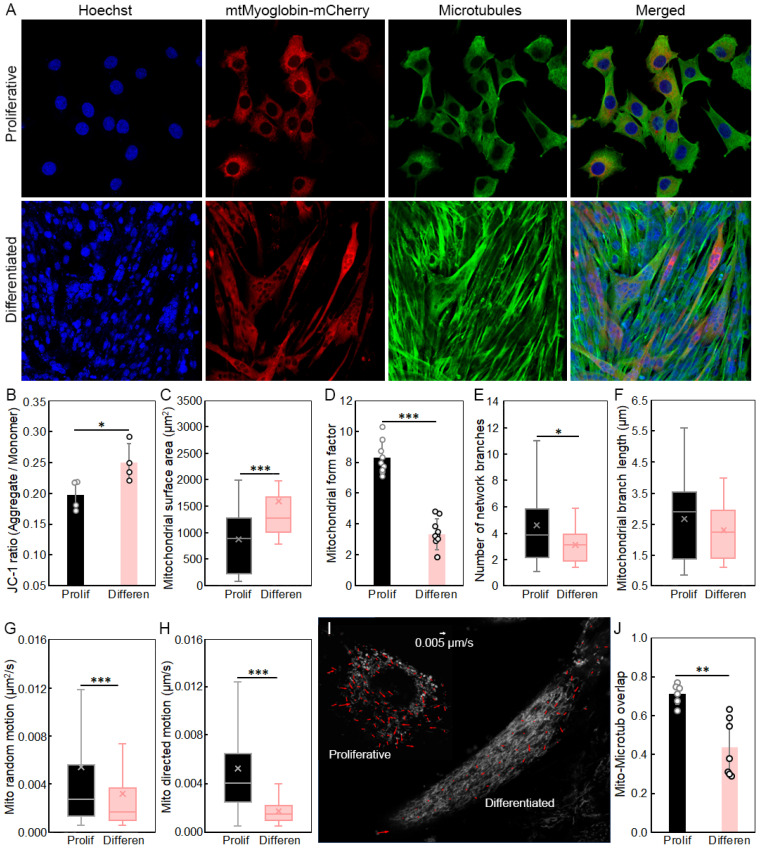
Changes in mitochondrial morphology and dynamics upon differentiation. (**A**), immunofluorescence staining of mitochondria-localized myoglobin-mCherry (red) and tubulin (microtubule marker, in green) in proliferative and differentiated C2C12 cells. Nuclei were counterstained with DAPI. Quantification of (**B**), mitochondrial membrane potential; (**C**), mitochondrial surface area; (**D**), mitochondrial form factor; (**E**,**F**), number of mitochondrial network branches and branch length; (**G**,**H**), mitochondrial random and directed motion. (**I**), mapping of mitochondrial-directed motion in a representative proliferative and a differentiated cell using spatiotemporal image correlation spectroscopy (STICS); the velocity scale arrows are 0.005 µm/s. (**J**), changes of mitochondria–microtubules (Mito-Microtub) association in proliferative (Prolif) and differentiated (Differen) C2C12 cells. Error bars represent standard deviations. For each two independent groups, statistical significance was defined using Mann–Whitney U test *p* < 0.05 (95% confidence level). Statistical significance was shown with stars on each plot: three stars (***) for *p*-value < 0.001, two stars (**) for *p*-value < 0.01 and one star (*) for *p*-value < 0.05. Each data point (empty circles) represents an independent experiment on a minimum of 20–30 cells.

First, we observed that mitochondrial distribution, while heterogenous, had consistent patterns across proliferative and differentiated cells (Figure 3A). In both cell types, mitochondria (shown in red) were distributed throughout the cytosol with a higher density in the perinuclear region (nucleus shown in blue). However, as shown in Figure 3B, transition from proliferative cells to differentiated cells was further accompanied by an increase in mitochondrial membrane potential, reflecting more bioenergetically efficient mitochondria.

**Figure A4:**
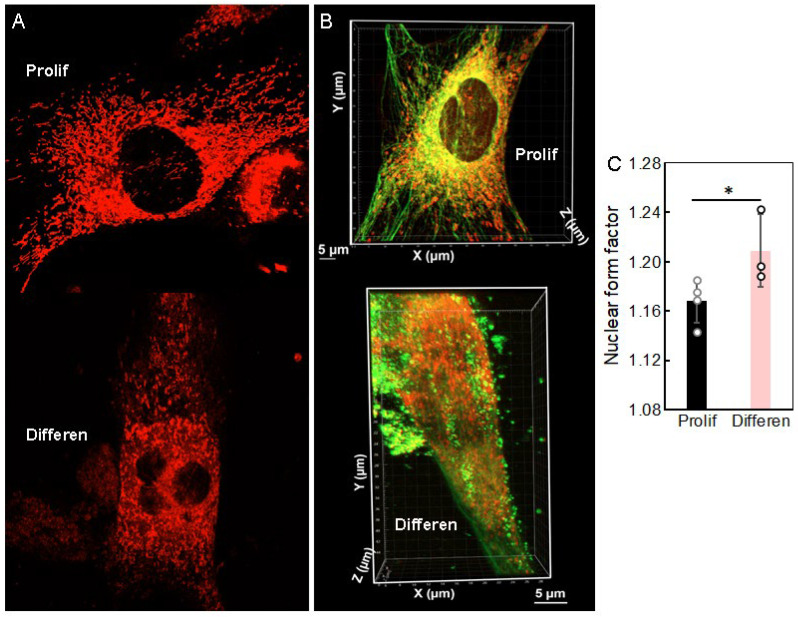
Mitochondria–microtubules association. (**A**), 2D fluorescence images of mitochondria in proliferative (Prolif) and differentiated (Differen) C2C12 cells. (**B**), 3D microscopy images of microtubules (green channel), mitochondria (red channel) in proliferative and differentiated C2C12 cells. (**C**), nuclear form factor in proliferative and differentiated cells. Error bars represent standard deviation. Statistical significance was shown with one star (*) for *p*-value < 0.05.

Characterization of mitochondrial morphology using the MiNA toolset revealed that differentiated cells contained a larger population of mitochondria with a greater surface area compared to proliferative myoblasts (Figure 3C). The increase in surface area is in agreement with our observation of increased citrate synthase (see Table A1), which has been shown to correlate with increased mitochondrial volume [27]. Calculation of the form factor in combination with MiNA revealed that differentiation promoted mitochondrial circularity (Figure 3D), possibly linked to increased mitochondrial biogenesis [28]. Form factor is a measure of mitochondrial shape and networked structures; a smaller form factor indicates less fused mitochondria and less branched networks. Although there were less mitochondrial network branches in differentiated cells (Figure 3E and Figure A4A in the Appendix A.5), the mean length of branched networks in differentiated cells was not significantly different from those in proliferative cells (Figure 3F).

Finally, we characterized changes in mitochondrial random and directed (flow) motions. Several time-series images of mitochondria in proliferative and differentiated C2C12 cells were recorded for the quantification of changes in mitochondrial random and directed (flow) motions. Correlation analyses of the fluorescence fluctuations in time, as recorded in the pixels of each image time-series (using temporal image correlation spectroscopy (TICS)), allowed us to determine the mitochondrial random motion throughout each cell (Figure 3G). Spatiotemporal image correlation spectroscopy (STICS) correlates fluorescence fluctuations in space and time, and it depends on both diffusion and flow directions of fluorescently tagged mitochondria [29,30], enabling us to measure and map the flow velocity vectors (Figure 3H,I). TICS and STICS results suggest that in contrast to highly mobile mitochondria in proliferative cells, mitochondria in differentiated cells undergo small-scale Brownian diffusion accompanied by some short-lived directed motions (Figure 3G–I).

As directed motion is largely dependent on microtubules [31,32,33], we investigated changes in mitochondria–microtubule associations. The Mander’s overlap coefficient was calculated from several z-stack of super-resolved fluorescence images. Representative microscopy images of mitochondria–microtubule associations are shown in Figure A4B in the Appendix A.5. As overlap coefficients may be difficult to predict in a heavily crowded area (due to accidental overlap) and to correct for random coincidences, the Mander’s overlap was recalculated by incorporating Costes randomization and thresholding as described in Section 3.9). While the changes we report are only meant to be qualitative, our analyses reveal that differentiated cells have reduced mitochondria–microtubule association, as evinced by a smaller overlap coefficient, compared to proliferative cells (Figure 3J).

In summary, these analyses indicate that during myogenic differentiation, mitochondria have bigger volume (and surface area), less branched networks and less association with microtubules.

## 3. Materials and Methods

### 3.1. C2C12 Cell Culture and Treatments

C2C12 cells were kept in high-glucose (HG, 4.5 g/L of glucose, 1 mM Sodium Pyruvate) Dulbecco’s modified Eagle’s medium (DMEM, Gibco, Grand Island, NY, USA) supplemented with 10% fetal bovine serum (FBS), and 1% penicillin-streptomycin solution (Mediatech Inc., Manassas, VA, USA). For imaging, cells were plated in μ-Slide 4 or 8 well chambers (Ibidi GmbH, Martinsried, Germany) at a density of 2.0–2.5 × 10^4^ cells/cm^2^. To induce muscle differentiation, C2C12 cells were grown to 70–80% confluency before switching to differentiation medium (2% horse serum in DMEM). Cells were kept in differentiation medium for up to 6 days with alternate days feeding. For glucose starvation studies, transfected C2C12 cells were kept in no glucose/no glutamine medium (GS, 1 mM sodium pyruvate) supplemented with 10% FBS and 1% penicillin-streptomycin solution for 24 h before imaging.

For mitochondrial perturbation experiments, a 2 μM rotenone inhibitor or 50 μM 2,4-dinitrophenol (DNP) mitochondrial uncoupler were added to C2C12 during the imaging.

### 3.2. Transfection with Myoglobin-mCherry O_2_ Probes

Myoglobin-mCherry is a Foster resonance energy transfer (FRET)-based sensor whose mean lifetime is sensitive to the O_2_-bound or O_2_-free state of the attached myoglobin, thus serving as a real-time equilibrium sensor. For O_2_ sensing, we used our previously reported myoglobin-mCherry lifetime fluorescence probe, of which we have different versions that target different spatial regions in the cell [11,34]. Herein, we use three versions of this probe. Briefly, the first, nontargeted “cytosolic” version of the pMyo-mCherry O_2_ probe was prepared by cloning the myoglobin gene from Physeter catodon (Sperm Whale, Addgene Plasmid pMB413a, #20058) into the pmCherry N1 vector (Clonetech, Mountain View, CA, USA). A 2-amino acid (Ser-Gly) spacer was inserted between the C-terminus of myoglobin and the N-terminus of mCherry to add flexibility and to prevent misfolding. For the second, mitochondrial-targeted probe, a sequence from the mouse mitochondrial transcription factor A (TFAM) was added to the pMyo-mCherry vector, to generate mtMyo-mCherry. TFAM has been shown to localize to mitochondria effectively [35]. Lastly, for the nuclear-targeted version, pMyo-mCherry was fused to three repeats of the nuclear localization signal of SV40 T antigen, to generate the nuclear-targeted O_2_ sensor (nlsMyo-mCherry). Validation of the localization of the three probes is provided in Figure A1 in the Appendix A.1.

C2C12 cells were transfected with each of the three versions of the O_2_ sensing probe in antibiotic-free medium using FuGENE^®^ HD transfection reagent (Promega Corporation, Durham, NC, USA), following the manufacturer’s instructions, with a final plasmid amount of ~100–200 ng per well. After 24 h, the transfection media was removed, and cells were incubated in fresh growth medium or switched to differentiation medium for an additional 24 h or 72 h before imaging (see immunofluorescence images of transiently transfected cells in Figure A1 in the Appendix A.1).

The BioTracker 488 Green Mitochondria Dye and Cell Viability Imaging Kit (both from Millipore Sigma, Sigma-Aldrich, Mannheim, Germany) were used for monitoring cell viability (Figure A2 in the Appendix A.2).

### 3.3. Assessment of Mitochondrial ROS, Membrane Potential and ATP in Bulk Populations

For the analyses of mitochondrial ROS (superoxide), mitochondrial membrane potentialbulk population measurements (no single cell) were used. For ROS measurements, cells were incubated with 1 µM MitoROS™ 580 (Cayman Chemical, Ann Arbor, MI, USA). MitoROS^TM^ 580 is cell-permeable and targets mitochondria. Once in the mitochondria, it can be oxidized, primarily by superoxide species, to become fluorescent [36]. For mitochondrial membrane potential, 5 μg/mL of JC-1 (tetraethylbenzimidazolyl-carbocyanine iodide) was used. JC-1 is a membrane-permeant dye that selectively accumulates in mitochondria. It is a ratiometric dye that shifts from green (monomer) to red fluorescence (multimer) as the membrane potential becomes more polarized [37,38,39]. Cells were incubated with either MitoROS^TM^ 580 or JC-1 for 30 min at 37 °C and 5% CO_2_ and then were washed at least three times before measurement. The ATP Bioluminescence Assay Kit CLS II (CREATIVE BIOMART^®^, Shirley, NY, USA) was used to assess total ATP and was used according to the manufacturer’s instructions. The reagents in this assay kit are not cell permeable; cells were lysed according to manufacturer’s instructions, and the result provides insight into total ATP. For MitoROS and ATP measurements, cells were additionally labeled with 10 μM Hoechst (Thermo Fisher Scientific, Rockville, MD, USA) to normalize the intensity profile based on the amount of DNA present in each well. The JC-1 assay is a ratio of red and green fluorescence, so Hoechst normalization was not used. Fluorescent signals were detected using a FLUOstar^®^ Omega, 5.70 R2 multi-mode microplate reader (BMG LABTECH, Cary, NC, USA) with absorption/emission filters of 355-20/460 nm for Hoechst, 485-12/590 nm for MitoROS, and Luciferase luminescence for ATP. Absorption/emission filters of 485-12/520 nm and 485-12/620 nm were used for the detection of JC-1 monomeric form with green fluorescence and J-aggregates with red fluorescence.

### 3.4. Modification of Mitochondrial O_2_ Consumption

Proliferative C2C12 cells, grown in monolayer, were kept in culture media with 2 μM rotenone inhibitor or 50 μM 2,4-dinitrophenol (DNP) mitochondrial uncoupler during imaging. Rotenone inhibits mitochondrial O_2_ consumption and DNP maximizes mitochondria O_2_ consumption. The effects of cellular glucose starvation on mitochondrial O_2_ level were investigated by culturing the transfected proliferative C2C12 cells in no glucose/no glutamine medium (GS, 1 mM sodium pyruvate), supplemented with 10% FBS and 1% penicillin-streptomycin solution for 24 h before imaging.

### 3.5. FLIM Set up with Controlled Imposed O_2_ Level

Two-photon FLIM was performed using a Leica SP5 confocal laser scanning microscope (Buffalo Grove, IL, USA) equipped with a tunable Chameleon Ti:Sapphire femtosecond laser (Coherent, UK) operating at 80 MHz with wavelengths set to 720, 780 and 880 nm for the excitation of NAD(P)H, Myo-mCherry and FAD^+^, respectively. The laser light was passed through a 685 nm long-pass dichroic mirror and directed to a Leica Plan-Apochromat 40×, 1.1 N.A. water immersion objective (laser power ≤ 7 mW at the objective). A 680 nm short-pass filter and a 560 nm long-pass dichroic mirror were used to, respectively, reduce the laser scattering and to split the NAD(P)H and FAD^+^ fluorescence toward two Leica hybrid photomultiplier detectors (HyD). NAD(P)H, FAD^+^ and mCherry signals were filtered using a 460/60 nm, a 552/57 nm and a 647/57 nm bandpass filter (Semrock BrightLine^®^, Rochester, NY, USA), respectively. The electrical pulse output from the HyD was directed into an SPC-150 photon counting card (Becker & Hickl, Berlin, Germany). Synchronization with the pixel, line and frame clock from the microscope scanning unit was used for image construction in time-correlated single photon counting (TCSPC) mode. Cells were imaged for 50–80 s (depending on the intensity). Image size was set to 256 × 256 (pixels)^2^, and TCSPC histograms were collected with 256 channels in a 12.5 ns time window.

A miniature CO_2_/O_2_/N_2_ gas mixing chamber (Bioscience Tools, San Diego, CA, USA) was mounted onto the microscope stage to keep the temperature at 37 °C and provide 5% CO_2_ and a stable imposed %O_2_ (*v*/*v*) of 18.5, 5.0 or 0.5% (the lowest %O_2_ attainable by our system) during imaging. On average, cells reached a stable pO_2_ within 45 min. A 250 μm diameter bare-fiber O_2_ sensor (long lived fluorescent bead quenched by O_2_) connected to an OxyLite, 1 Channel monitor (Optronix Ltd., Oxford, UK) was used to measure the media-imposed external pO_2_ (in mmHg) at the bottom of the Ibidi chambered dishes.

### 3.6. Measurements of Intracellular pO_2_, Free/Bound NAD(P)H and Redox Ratio

Lifetime imaging recordings of the FRET sensor Myo-mCherry, and of the auto-fluorescent metabolic co-factors NAD(P)H and FAD^+^ were used to assess the cellular metabolic state at different imposed O_2_%. The fluorescence lifetime decays of Myo-mCherry, NAD(P)H and FAD^+^ were obtained by a double-exponential decay model in SPCImage (Becker & Hickl GmbH) at optimized goodness of fit (*χ_r_*^2^). The pre-exponential factors *a*_1_% and *a*_2_% for NAD(P)H and FAD^+^ were generated via amplitude weighting for each pixel to calculate the free/bound NAD(P)H (*a*_1,NAD(P)H_ %/*a*_2,NAD(P)H_ %) and the fluorescence lifetime based redox ratio (FLIRR; *a*_2,NAD(P)H_%/*a*_1,FAD_%) [12]. If natural lifetime is constant, *a*_1_ and *a*_2_ represent the population fraction of fluorophores with shorter (*τ*_1_) and longer (*τ*_2_) lifetimes, respectively.

For intracellular pO_2_ measurements, the mean lifetime of Myo-mCherry was obtained for each respiring and non-respiring (treated with rotenone) single cell, and averaged across multiple cells collected in 3 independent experiments. We routinely used rotenone-treated cells because we did not see any significant differences between their lifetime values and those obtained for mitochondrial DNA-depleted non-small cell lung cancer (A549-*ρ*^0^) cells and SCO2^−/−^ cells [34,40]; SCO2^−/−^ cells had genetically disrupted respiration via deleting SCO2. Then, the resulting lifetime values (*τ*(pO_2_)) obtained for the non-respiring cells were plotted against the media-imposed external pO_2_ and a hyperbolic curve was fit to the data using the Curve Fitting Toolbox in MATLAB R2020b (The MathWorks Inc., Natick, MA, USA) as described previously [11]:

(1) $$\tau \left(pO_{2}\right)=\left(\tau_{max}-\tau_{min}\right)\frac{pO_{2}}{K+pO_{2}}+\tau_{min}$$

where *τ_max_* and *τ_min_* are the longest and shortest average lifetime for Myo-mCherry at normoxia and hypoxia, respectively. *K* is a fitting parameter directly related to the affinity of myoglobin for O_2_.

The intracellular pO_2_ in rotenone-treated proliferative cells (incapable of significant O_2_ consumption) is assumed to be equivalent to the media-imposed pO_2_. Therefore, *τ*(*pO*_2_) values for the treated cells were used as a reference for the Myo-mCherry lifetime at the pO_2_ level present in the solution. The *τ*(*pO*_2_) values of actively respiring C2C12 cells were then compared to those obtained for the reference. Rearranging Equation (1), the effective pO_2_ at each lifetime value was back calculated for any respiring cells by fixing the *τ_max_, τ_min_* and *K* to 1.28 ns, 0.914 ns and 7.56 (all values obtained from the rotenone data).

Finally, pseudo color mapping of pO_2_ and free/bound NAD(P)H in the intracellular environment was obtained using SPCImage as described in Ref. [11].

### 3.7. Measurements of Mitochondrial Directed and Random Motions

Mitochondria were labeled with 200 nM MitoTracker^®^ Green (Molecular Probes^®^, Eugene, OR, USA) and time-series images were acquired using a commercial Zeiss 880 Confocal laser scanning microscope (Jena, Germany) equipped with a 63×/1.4 NA Oil immersion objective. The beam from a multiline argon laser emitting at 488 nm was directed onto the sample using a dual band dichroic mirror, MBS 488/594. The green fluorescence collected from the sample was directed toward a Zeiss PMT and was spectrally filtered by selecting a 490−531 nm bandpass filter. The individual frames (512 × 512 pixels, 70 frames) of each series were collected with a pixel dwell time of 2.55 μs and no added time delays between the sequential frames.

TICS and STICS analyses were performed using MATLAB R2022b (The MathWorks Inc., Natick, MA, USA) routines adapted from the works of Wiseman Research group, McGill University [14,41]. These fluorescence-based microscopy techniques are appropriate for the study of molecular diffusion and flow on time scales ranging from microseconds to milliseconds. The STICS spatiotemporal autocorrelation functions were performed for different sub-regions of the images. The spatiotemporal component of these correlation functions was averaged and fitted by nonlinear least squares. For the same sub-regions of the images, the TICS temporal autocorrelation functions were calculated to obtain the magnitude of random motion. Each image correlation analysis was performed at least on 10 cells of the same type with at least 30 distinct sub-regions for each cell.

### 3.8. Analyses of the Mitochondrial Morphology and Form Factor

With the same Mitotracker Green labeled images used for the TICS and STICS analyses, we performed additional analyses using the MiNA ImageJ 1.54 p macro tool [42]. This toolset uses preprocessing options including the filters unsharp mask and median, along with enhancing local contrast (CLAHE), to increase the contrast of the labeled mitochondria in the image. Then, the image is converted into binary, followed by conversion to a skeleton that represents the features in the original image using a wireframe of lines one pixel wide. Each pixel of the skeleton is then classified and spatially related and defined to measure the length of each branch and the number of branches in each skeletonized feature.

Additionally, the same skeletonized images were processed with ImageJ’s built in analysis tool “Analyze Particles” to resolve perimeters (*p*) and areas (*a*) of each skeleton. Then, these parameters were used to calculate the form factor defined as [43]:

(2) $$FF=n^{-1}\sum^{n}\frac{p^{2}}{4\pi a}\;$$

### 3.9. Changes of Mitochondria–Microtubule Association upon Differentiation

Mitochondria and microtubules in live proliferative and differentiated cells were labeled with MitoTracker^TM^ Red FM (Invitrogen, Eugene, OR, USA) and ViaFluor^®^ 488 Microtubule staining Kit (Biotium, CA, USA). Three-dimensional live cell images were acquired using a Zeiss 980 Confocal laser scanning microscope equipped with Airyscan (both Jena, Germany). This microscope can resolve structures ~120 nm apart in the lateral dimension and ~350 nm apart in the axial dimension. The beams from multiple lasers emitting at 488 and 568 nm were directed onto the sample using a Plan-Apochromat 63×/1.4 NA Oil immersion objective. The fluorescence signals collected from the sample were directed toward proprietary Zeiss Photomultiplier Tubes and were spectrally filtered by selecting a 380–735 nm for ViaFluor 488 and a 600–630 nm filter for MitoTracker Red. Then, for the colocalization between mitochondria and microtubules, Mander’s overlap coefficient was recalculated by incorporating Costes randomization and thresholding using BIOP/JACoP toolset in ImageJ.

### 3.10. Statistical Analyses

Multiple cells and/or plate wells were measured in each condition over different days in at least three independent experiments (shown as individual data points); each experiment includes at least 10–15 cells and a total number of at least 30 cells. Bar charts with individual data points and standard deviations were used to represent the mean values. Box plots were also created to visually compare the data for mitochondrial directed (flow) and random motions with large standard deviations; means and anomalies were created at *p* < 0.05 (or 95% confidence level). Mann–Whitney U tests or *t*-tests were carried out using SPSS 14.0 software (IBM, Chicago, IL, USA) to evaluate whether the values in each two independent groups were significantly different from each other. Statistical significance was defined at *p* < 0.05 (95% confidence level) and was shown with stars on each plot: three stars for *p*-value < 0.001, two stars for *p*-value < 0.01 and one star for *p*-value < 0.05.

## 4. Conclusions

Current studies investigating the role of mitochondria in cell differentiation use bulk population-level measurements. Population-averaged assays are powerful tools in biology, enabling the identification of components and interactions within complex metabolic, signaling and transcriptional networks. Such assays, however, implicitly assume that cell populations are homogeneous.

Heterogeneity is often observed in biology reflecting genetic, epigenetic and environmental factors, which highlights the necessity to study the biochemical and physiological characteristics of cells and their environment at single-cell resolution.

Single-cell-based imaging and analysis in this study provide insights into the heterogeneity of metabolism and dynamics of proliferative and differentiated C2C12 cells. We used several imaging techniques to spatially resolve and quantify mitochondrial pO_2_ and metabolic changes at subcellular resolution. We show that myogenic differentiation requires a metabolic switch to support the increased energetic demand of contractile muscle. Our techniques revealed that myogenic cells shift from a highly glycolytic state in proliferating conditions to relying on OXPHOS upon differentiation. In addition, our analyses at the single-cell level revealed heterogeneity in the intracellular pO_2_ level, free/bound NAD(P)H ratio, FLIRR (or metabolic signature) and mitochondrial dynamics both between different cells in a population (Figure A3) and within the same cell’s intracellular environment (Figure 1B and Figure 2A,B). This heterogeneity was more pronounced among proliferative cells kept in high-glucose/regular media (Figure A3). Within this population, O_2_ consumption and OXPHOS (correlating with lower cytosolic pO_2_) were higher in cells in the G2/M phase, while cells relied more on glycolysis for their energy needs in the G1 phase. 

We also provided evidence that mitochondria are the primary sites of O_2_ consumption and mitochondrial oxygenation in both proliferative and differentiated C2C12 cells alters the levels of O_2_ at the nuclear core.

Finally, we showed that the multifaceted involvement of mitochondria in C2C12 differentiation is accompanied by significant changes in mitochondrial dynamics (both morphology and motion). While mitochondrial surface area increased, a significant decrease in mitochondrial network branches, form factor, motion (directed and random) and association with microtubules was detected upon differentiation.

## Data Availability

Data are available in figshare and on request.

## Abbreviations

The following abbreviations are used in this manuscript:

OXPHOS	Oxidative phosphorylation
ATP	Adenosine triphosphate
O_2_	Oxygen
pO_2_	Oxygen partial pressure
Myo-mCherry	Myoglobin-mCherry
HG	High-glucose media
GS	Glucose-starved media
FLIM	Fluorescence lifetime imaging microscopy
FRET	Förster resonance energy transfer
NAD(P)H	Nicotinamide adenine dinucleotide
FAD^+^	Flavin adenine dinucleotide
MiNA	Mitochondrial Network Analysis
ROS	Reactive oxygen species
TICS	Temporal image correlation spectroscopy
STICS	Spatiotemporal image correlation spectroscopy
DMEM	Dulbecco’s modified Eagle’s medium
FBS	Fetal bovine serum
DNP	2,4-dinitrophenol
HyD	Hybrid detector
Rot	Rotenone
FAO	Fatty acid oxidation
GLUT1	Glucose transporter protein type 1
HK1	Hexokinase 1

## Appendix A

### Appendix A.2. Cytotoxicity Studies

Brightfield images of the proliferative cells were collected over several days to see their progression toward the differentiation (Figure A2A). To test for cytotoxic effects of transfecting cells with the O_2_ sensor constructs, transfected C2C12 cells were labeled with 100 nM BioTracker 488 Green Mitochondria Dye (Millipore Sigma, Burlington, MA, USA) for 15 min, and the fluorescence signal was detected using a FLUOstar^®^ Omega multi-mode microplate reader (BMG LABTECH) with absorption/emission filters of 485-12/520 nm (Figure A2B). Viability Imaging Kit (Millipore Sigma, Sigma-Aldrich, Germany) was also used to study cell viability in both proliferative and differentiating conditions (Figure A2C). The percentage of viable and dead cells was determined using the dyes provided in the Assay Kit with absorption/emission filters of 485-12/520 nm and 544/620 nm, respectively. C2C12 cells were additionally labeled with 10 μM Hoechst (Thermo Fisher Scientific) to normalize the intensity profile of both assays based on the amount of DNA present in each well. We did not detect any significant sign of cytotoxicity or decrease in cell viability due to the transfection or upon differentiation. The transfection efficiency (Figure A2D) was additionally calculated from the fluorescence images shown in Figure A1A,B.

### Appendix A.3. Heterogeneity of Intracellular Oxygenation Level and Metabolic Redox Ratio

Heterogeneity of the intracellular pO_2_ was investigated using single-cell based measurements of compartmental pO_2_ (cytosolic, mitochondrial and nuclear). Scatter plots of individual proliferative cells cultured in standard, high-glucose (HG) conditions, as well as glucose-starved (GS) conditions, treated with the mitochondrial uncoupler DNP and upon differentiation, are shown in Figure A3A. The relationship between the heterogeneity of intracellular oxygenation level and cell cycle was also investigated by plotting cytosolic pO_2_ versus Hoechst intensity in Figure A3B. The fluorescence intensity of Hoechst increases upon binding to double-stranded DNA, allowing for the separation of cells in different phases of the cell cycle. During the G2 phase before mitosis, Hoechst intensity increases due to the doubling of DNA content. The G1 phase is the least stained because these cells have the least DNA (2N) compared to cells after S-phase DNA replication (4N DNA). The results showed that the heterogeneity in cytosolic pO_2_ is due to the presence of cells throughout the cell cycle (G1 at top and G2/M at the bottom). While mitochondrial respiration and OXPHOS increased (less intracellular pO_2_) in the G2/M phase of the C2C12 cell cycle, the cells relied more on glycolysis for their energy needs during the G1 phase.

Figure A3C,D shows individual data points representing metabolic heterogeneity and reflecting measurements of free/bound NAD(P)H and metabolic redox ratio FLIRR. In agreement with Figure A3A, the highest heterogeneity was measured in proliferative C2C12 cells cultured in standard, HG conditions, suggesting that glycolysis/OXPHOS change is accompanying the cell cycle.

### Appendix A.4. Mass Spectrometry of α-, β- Subunits ATP Synthase, Citrate Synthase, Vimentin and Desmin

As shown in Table A1, whole-cell proteomics of proliferative and differentiated C2C12 cells were performed by mass spectrometry at Poochon Scientific (Frederick, MD, USA). From these data, abundances of *α*- and *β*- subunits of ATP synthase, citrate synthase, vimentin and desmin were extracted and normalized to total protein counts (based on the spectral count for a given protein, normalized to the total spectral count in each data set) and reported as normalized abundance. Biological replicates were processed for the replicating myoblasts as an indication of reproducibility, with single values for the differentiated myotubes.

The increase in ATP synthase (mitochondrial) subunits is consistent with increased mitochondrial oxidative phosphorylation (OXPHOS)-driven ATP synthesis in differentiating cells. Citrate synthase is a protein in the matrix of the mitochondria, which performs the first step in the citric acid cycle. Vimentin expression, abundant in mesenchymal, migrating, proliferative cells is downregulated, in favor of the muscle-specific intermediate protein, desmin, whose expression is increased.

**Table A1:** Mass spectrometry proteomics-derived abundances of ATP synthase, citrate synthase, vimentin and desmin. These results are qualitative validation of differentiation, not quantitative determination of the exact protein expression levels.

	Protein Abundance, Counts × 10^−9^		
Protein	Proliferative Cells Ave (std) n = 5	Differentiating Cells n = 1	Differentiating/Proliferative
ATP synthase *α*	5.7 (0.9)	17	3
ATP synthase *β*	6.6 (1)	17	2.6
Citrate synthase	0.08 (0.04)	2.0	25
Vimentin	112 (5.4)	42	0.4
Desmin	8.8 (1.6)	17	1.9

### Appendix A.5. Changes of Mitochondria–Microtubule Association and Microtubules Morphology upon Differentiation

Mitochondria were stained with MitoTracker Red and microtubules were labeled with ViaFluor Live Cell Microtubules 488 to investigate changes of mitochondrial morphology and their overlap with microtubules. Representative 2D microscopy images of mitochondria (red channel) and 3D images of mitochondria–microtubules (green channel) association in proliferative and differentiated C2C12 cells are shown in Figure A4A,B, respectively. In agreement with Ref. [44], nucleus size in the differentiated cells decreased, and form factor increased (Figure A4C).
